# Antipsychotic co-medication and treatment response to rTMS and iTBS in depression: Data from clinical records from two independent clinical sites

**DOI:** 10.1177/02698811251413510

**Published:** 2026-02-23

**Authors:** Amelie Völkel, Joshua Tritsch, Marco Poli, Katharina Kerkel, Alkomiet Hasan, Berthold Langguth, Andreas Reissmann, Wolfgang Strube

**Affiliations:** 1 Faculty of Medicine, Department of Psychiatry, Psychotherapy and Psychosomatics, University of Augsburg, Bavaria, Germany; 2 DZPG (German Center for Mental Health), Partner Site Munich/Augsburg, Germany; 3 Department of Psychiatry and Psychotherapy, University of Regensburg, Germany

**Keywords:** transcranial magnetic stimulation, theta burst stimulation, depression treatment, noninvasive brain stimulation, antipsychotic augmentation

## Abstract

**Rationale:**

Transcranial magnetic stimulation (TMS) is a well-established, noninvasive method for modulating cortical activity and has demonstrated efficacy in the treatment of depression. This study investigated potential interaction effects between antipsychotic augmentation and TMS efficacy using clinical records from two independent sites. In addition, exploratory analyses examined the association between resting motor threshold (RMT) and treatment outcomes.

**Methods:**

We analyzed naturalistic data from patients with depressive symptoms treated at the TMS outpatient departments in Augsburg (*n* = 53) and Regensburg (*n* = 120). Depressive symptom severity was assessed using the Beck Depression Inventory, Hamilton Rating Scale for Depression-17, and Major Depression Inventory at baseline, during treatment, and upon treatment completion. Patients were grouped according to whether or not they received antipsychotic augmentation. Group comparisons were conducted using Mann–Whitney *U* tests. For secondary analyses, scatter plots explored associations between baseline RMT and improvements in depressive symptoms, and correlation coefficients were calculated.

**Results:**

At the Augsburg site, 2 weeks after treatment initiation, patients not receiving antipsychotic augmentation exhibited significantly greater improvement in depressive symptom severity. However, differences between groups were not significant at treatment initiation or upon completion. Further, RMT analysis at Augsburg indicated a numerical but nonsignificant correlation with treatment outcome. At the Regensburg site, no consistent interaction effects were observed, and RMT analyses revealed nonsignificant correlations.

**Conclusions:**

Taken together, these findings suggest that TMS can remain effective in patients receiving concomitant antipsychotic augmentation alongside antidepressant and TMS treatment. Future research could usefully examine whether TMS remains effective in patients receiving different kinds of antipsychotic medication.

## Introduction

Transcranial Magnetic Stimulation (TMS) is a noninvasive brain stimulation technique that can be used to modulate brain activity and investigate brain-behavior relationships. It has gained considerable attention in both research and clinical settings for its ability to influence cortical physiology and potentially treat neurological and psychiatric conditions, particularly depressive disorders (Berlim et al., 2013; De Risio et al., 2020; Dell’Osso et al., 2011; George et al., 2013; Gross et al., 2007). Both rTMS and the more recently developed intermittent theta burst stimulation (iTBS; Blumberger et al., 2018) can be subsumed as TMS applications and have been established as treatment options alongside pharmacotherapy and psychotherapy in major depressive disorder. The aim of current TMS research is to further optimize treatment parameters and to understand the underlying mechanisms to create the best possible treatment conditions.

Against this background, one critical focus of TMS research lies in determining the optimal treatment duration. Both rTMS and iTBS for depression are typically administered over a period of 4–6 weeks, with 20–30 treatment sessions conducted during this time (Pohar and Farrah, 2019). Evidence suggests that the effectiveness of these TMS techniques primarily depends on the number of sessions rather than the overall duration of the treatment (Galletly et al., 2012; Schulze et al., 2016). In a large-scale registry study, Hutton et al. (2023) concluded that patients who received fewer than 30 sessions of TMS treatment had worse endpoint outcomes than patients who received more sessions. Symptom reduction was greatest in the group that stopped treatment after 36 sessions. In clinical practice, booster sessions may be scheduled after the main treatment cycle if symptoms recur or do not fully resolve, and mixed results have been shown for these strategies as potential influencing factors for TMS efficacy (Lew et al., 2025; Razafsha et al., 2023). Accordingly, the number of treatment sessions is discussed as an important influencing factor in terms of effective dosage of TMS applications. In this context, other TMS-related parameters have increasingly gained attention as possible influencing factors for effective dosing. The resting motor threshold (RMT) in particular has gained attention in recent years, not only due to its potential variability before and during rTMS and iTBS treatments (Bourla et al., 2023; Nordmann et al., 2015; Ter Braack et al., 2019) but also because of the question of whether it influences treatment response (Lee et al., 2025).

Finally, TMS applications are rarely used as monotherapy in clinical settings, raising questions about the interactions with other therapeutic approaches, such as pharmacotherapy and psychotherapy. Concurrent use of medication has been shown to influence the outcome of TMS treatments. For example, the coadministration of GABA-A receptor positive allosteric modulators (e.g., Diazepam and Lorazepam) appears to diminish the effects of rTMS, whereas other psychopharmacological agents may suggest additive effects (Deppe et al., 2021; Hunter et al., 2019; Kochanowski et al., 2024). In particular, the neurotransmitter dopamine has become the focus of neurobiological rTMS research, as positron emission tomography (PET) studies have shown that rTMS of certain brain regions, such as the motor cortex, the dorsolateral prefrontal cortex (DLPFC), as well as the dorsomedial prefrontal cortex (DMPFC), influences dopamine activity in corresponding brain areas (Cho and Strafella, 2009; Strafella et al., 2003). At the same time, the study by Monte-Silva et al. (2011) provided evidence that dopamine D2 antagonists such as sulpiride in common clinical doses can suppress the inhibitory and excitatory effects of neurophysiological rTMS paradigms in healthy subjects. These findings suggest that dopamine D2 receptor antagonism may interfere with the neuroplastic effects of rTMS treatments. This is supported by PET studies demonstrating dopamine release following rTMS over frontal areas (Cho and Strafella, 2009; Strafella et al., 2003). Conversely, dopamine and norepinephrine multimodal agents such as lisdexamfetamine or methylphenidate have been associated with enhanced clinical response to rTMS (Hunter et al., 2019). This research formed a basis for previous studies exploring the impact of medications in naturalistic settings. Schulze et al. (2017) examined 105 patients with treatment-resistant depression in a clinical setting and found, contrary to their expectations, that patients taking D2/5-HT2 receptor antagonists or D2/5-HT1A partial agonists tended to benefit more from TBS over the DMPFC. The authors discussed different stimulation parameters, the inclusion of clinical populations instead of healthy subjects, and different outcome measures compared to the preclinical studies (motor evoked potentials vs depression values) as possible explanations for their findings. Unequivocally, results from a naturalistic sample of 299 patients from Hebel et al. (2020) supported that patients receiving D2/5-HT2 receptor antagonists or D2/5-HT1A partial agonists as a part of their antidepressive treatment plan showed significantly lower improvements in depressive symptoms compared to patients without medication with these mechanisms of action.

Given these numerous potential influencing factors on TMS effectiveness, respectively, our register-based analysis sought to investigate the interaction between D2/5-HT2 receptor antagonists and D2/5-HT1A partial agonists and TMS, as well as the potential impact of RMT on treatment response in a naturalistic patient sample.

## Methods

### Study design

#### Augsburg

The reported results are based on routine data from patients from two independent sites. First, the TMS outpatient clinic of the Department of Psychiatry, Psychotherapy and Psychosomatics of the University Hospital Augsburg (Germany) was analyzed, where a total of 61 in- and out-patients were treated with rTMS or iTBS over the left DLPFC between November 2021 and July 2024, 53 of whom were included in the study (see below). Patients received treatment 5 times a week for 4 weeks, for a total of 20 sessions at a stimulation intensity of 80% of the individual RMT, which was determined via both the Rossini–Rothwell–Method and, whenever possible, an adaptive threshold hunting method (Ah Sen et al., 2017; Rossini et al., 1994). When the two methods yielded different RMT values, the mean of the two values was determined and used. The Beam-F3-Method was used to determine the stimulated target region (Beam et al., 2009). For rTMS, a MagVenture R30 stimulator with a water-cooled 70 mm Cool-B70 coil (MagVenture/Willich/Germany) was used. The protocol consisted of 40 trains of 10 Hz stimulation, each lasting 5 seconds with an inter-train interval (ITI) of 20 seconds, totaling 2000 pulses per session (session duration: 800 seconds). Stimulation intensity was set to 80% RMT. iTBS was delivered with a PowerMAG Clinical 100 device and an air-cooled 70-mm figure-of-eight aCool coil (Mag&More/Munich/Germany), according to the established protocol (3 pulses at 50 Hz at a 5 Hz burst frequency), with 2-second trains repeated every 10 seconds (ITI = 8.16 seconds) for a total of 600 pulses in 192 seconds. The stimulation intensity was set to 80% RMT. During stimulation with both rTMS and iTBS, the coil was pointed toward the patient’s nasion at a 45° angle to the midline and tilted tangentially to the head (Lisanby et al., 2001; Wobrock et al., 2015). Participants were retrospectively grouped into the augmentation group if, at the start of their stimulation series, D2/5-HT2 receptor antagonists or D2/5-HT1A partial agonists were included in their medication regimen, irrespective of individual dosages or treatment goals (antidepressant augmentation, mood stabilization, sleep- or antipsychotic therapy).

#### Regensburg

The second site analyzed a cohort of patients treated at the Regensburg Center for Neuromodulation, situated at the outpatient clinic of the Department of Psychiatry and Psychotherapy of the University of Regensburg at the medbo Bezirksklinikum Regensburg (Germany). Between August 2016 and December 2023, 1395 in- and out-patients were treated with iTBS or rTMS and were further assessed for eligibility regarding the current study. A final sample of 121 patients was included for the present study analyses (see below).

Administered treatments consisted of 5 stimulation sessions per week for 6 weeks, for a total of 30 sessions. RMT was determined using the Rossini–Rothwell–Method (Rossini et al., 1994). The target region F3 was identified according to the international 10–20 system using a perforated EEG cap with pre-marked electrode positions. The cap was individually fitted to each participant’s head size and positioned such that the marked Cz and Fpz locations aligned with the individually determined anatomical landmarks. MagVenture MagPro X100 stimulation devices with MagOption modules were used in conjunction with water-cooled 65 mm Cool-B65 coils (MagVenture/Willich/Germany). TMS pulses were applied using the biphasic waveform and the default (i.e., “Normal”) current direction setting of the MagPro devices.

For the rTMS protocol, 40 trains of TMS pulses at 20 Hz were applied for 2.5 seconds and an ITI of 25 seconds, using a treatment intensity of 110% RMT (pulses per session: 2000, session duration: 1080 seconds).^1^ The iTBS protocol consisted of the standard protocol outlined above (see “Augsburg”), applying a treatment intensity of 120% RMT. Both for rTMS and iTBS treatments, maximum stimulation intensity was limited to 60% of the maximum stimulator output of the MagPro devices. During treatment, the TMS coil was oriented at a 45° angle relative to the midsagittal line (with the handle point backward), tilting it tangentially to the head. Patients were retrospectively grouped into the augmentation group when they received D2/5-HT2 receptor antagonists or D2/5-HT1A partial agonists as part of their medication regimen during the rTMS/ iTBS treatment phase (see “Augsburg”).

### Participants

To be included in the analysis, patients from Augsburg and Regensburg had to be between 18 and 70 years old and have a primary diagnosis of depression (ICD-10: F31–F33) (*World Health Organization, 2019*). In addition, a completed depression rating scale (Beck Depression Inventory (BDI), Hamilton Rating Scale for Depression (HAMD), or Major Depression Inventory (MDI)) at baseline had to be available, with no more than two missing item responses per instrument. Those instruments are used as part of the routine clinical care. Patients were required to have received at least 80% of planned treatment sessions, with no interruptions (e.g., Christmas holidays) during the treatment phase. For the duration of treatment, full information regarding medication intake had to be available.

Patients with serious somatic illnesses such as epilepsy or severe cardiovascular disease were excluded from all analyses, as were patients with diagnosed tinnitus aurium.

After a medical consultation with an attending physician, all patients, regardless of their study inclusion, had to provide the ability to comprehend and sign an informed consent to their treatment and the pseudonymized storage and evaluation of their data.

The evaluations at both study sites were approved by the respective local ethics committee (University of Munich (19-672 3), University of Regensburg (22-2958-104)).

#### Augsburg

Of the 61 treated patients, 53 met all inclusion criteria. Patients were excluded based on missing ratings at baseline (*n* = 8) and duplicate entries in our database (*n* = 1). Because of missing data at specific timepoints, some analyses were based on reduced subsamples, which are further specified along their respective tests.

#### Regensburg

To qualify for subsequent screening, the initial pool of 1395 patients was checked for the availability of symptom information data before, during, and after the TMS treatment. Contrary to Augsburg, the standard procedure at the Regensburg Center for Neuromodulation involves only pre- and post-treatment symptom evaluations. Because of this, the number of eligible patients was drastically reduced to 200 patients, who had planned interim symptom evaluations and had received at least 80% of the planned treatment sessions.

The final pool of eligible patients was further reduced to 121 patients, since there were cases with missing data from (at least one of the) clinical scales or from medication intake (*n* = 51), other primary diagnoses (*n* = 8) or comorbid tinnitus aurium (*n* = 8), repeated TMS treatment (*n* = 2) as well as interruptions of the treatment phase (*n* = 10).

### Outcomes

#### Augsburg

The primary outcome in this retrospective exploratory analysis was the difference in BDI scores between patients being treated with and without D2/5-HT2 receptor antagonists or D2/5-HT1A partial agonists 2 weeks (T1) and 4 weeks (T2) after baseline (T0). The correlation between the RMT at baseline and the absolute improvement in BDI scores from baseline to post-treatment was explored as part of a secondary, exploratory analysis.

#### Regensburg

The primary outcomes were the differences in MDI and HAMD scores between patients being treated with and without D2/5-HT2 receptor antagonists or D2/5-HT1A partial agonists 3 (T1*) and 6 weeks (T2*) after baseline (T0*). The secondary outcomes were the correlations between RMT at baseline and the absolute improvements in HAMD and MDI scores 3 and 6 weeks after baseline.

### Statistical analysis

All tests were performed two-tailed, with a significance threshold set at α = 0.05. Continuous variables were assessed for normality both visually (histograms and Q-Q plots) and statistically (using the Shapiro–Wilk test). Data following a Gaussian distribution are reported as mean ± their standard deviation (SD), while non-Gaussian distributed data are reported as median with their interquartile range (IQR, 25th–75th percentile). Sociodemographic characteristics for all participants are reported grouped by treatment with D2/5-HT2 receptor antagonists or D2/5-HT1A partial agonists. Between-group differences were tested with Mann–Whitney *U* tests for continuous variables and Chi-squared tests for categorical variables, as the data did not meet the assumptions of normality described above. To verify the overall treatment effect, a Friedman test with post hoc Wilcoxon signed-rank tests was performed. Because our study only explored differences between two groups, direct pairwise Mann–Whitney *U* tests were performed in place of a Kruskal–Wallis test to analyze between-group differences at all 3 timepoints. Bonferroni–Holm correction was applied where applicable to adjust for multiple comparisons. Due to the small Augsburg sample and the exploratory nature of this study, we opted against more complex nonparametric omnibus tests to explore our primary hypothesis. For the secondary analysis, scatterplots with RMT at baseline as the independent and absolute improvements on the study sites’ respective ratings scales as the dependent variable were constructed and appropriate correlation coefficients were calculated to quantify these relationships.

#### Augsburg

The data were analyzed using the R statistical software (v.4.2.2; R: R Core Team, 2025). For participants with one or two missing BDI items, mean imputation was used by calculating the average of completed items and multiplying by 21, as outlined in the BDI-II manual (Beck et al., 1996).

#### Regensburg

Data analysis was conducted using IBM SPSS Statistics (Version 29.0; IBM Corp., 2022). Missing values in item-level data from the HAMD and MDI scales were handled using multiple imputation. Five imputations were generated using fully conditional specification with automatic method selection. Upper and lower bounds were defined based on each item’s response range (e.g., 0–4, 0–2, or 0–5). Only item-level values were imputed; no total scores or transformed variables were included. Reported results include the observed ranges in test statistics and p-values across the imputed datasets. The secondary analysis was conducted on the original sample (i.e., without the use of the imputed datasets).

## Results

### Sociodemographic variables

#### Augsburg

Patients taking D2/5-HT2 receptor antagonists or D2/5-HT1A partial agonists were significantly older compared to patients receiving no D2/5-HT2 receptor antagonists or D2/5-HT1A partial agonists (*p* = 0.045). No other significant differences were observed (all *p* > 0.100; see Table 1 for details).

**Table 1.** Baseline sociodemographic characteristics of the Augsburg patient sample.

Characteristics	Receiving augmentation		Statistics^c^
	No, *n* (%)	Yes, *n* (%)	
Total^a^	26 (50.0)	27 (50.0)	
Age^b^	34 (29–38)	50 (30–56)	*W* = 238, *p* = 0.045
Sex			*p* > 0.999
Male^a^	13 (52.0)	14 (52.0)	
Female^a^	13 (48.0)	13 (48.0)	
Taking MRIs^a,e^	19 (73.0)	23 (85.0)	*p* = 0.455
Taking GABA-A PAM (Z)^a,f^	1 (3.8)	3 (11.0)	*p* = 0.631
Taking GABA-A PAM (BZD)^a,g^	2 (7.7)	4 (15.0)	*p* = 0.701
Taking GA/ICA^a,h^	5 (19.0)	4 (15.0)	*p >* 0.950
Stimulation protocol^a^			*p* = 0.889
TBS^a^	12 (46.0)	14 (52.0)	
rTMS^a^	14 (54.0)	13 (48.0)	
Resting motor threshold^c^	40 (35–46)	39 (35–47)	*W* = 342, *p* = 0.873
BDI at baseline^b^	26 (19–33)	32 (21–37)	*W* = 281, *p* = 0.212

a *n* (%).

b Median (Q1–Q3).

c Mean ± SD.

d Chi-squared test for categorical variables, Wilcoxon-test for continuous variables.

e Monoamine Reuptake Inhibitors. Includes drugs formally known as Antidepressants.

f GABA-A Positive Allosteric Modulators. Includes drugs formally known as *Z*-drugs (e.g., zopiclone, zolpidem), used as hypnotics.

g GABA-A Positive Allosteric Modulators (Includes drugs formally known as benzodiazepines).

h Glutamate and Ion Channel Agents. Includes drugs formally known as mood stabilizers.

#### Regensburg

For the Regensburg sample, patients receiving antipsychotic medication differed significantly from those without such medication in terms of several baseline characteristics. Specifically, there were group differences in sex (*p* = 0.015), antidepressant use (*p* < 0.001), benzodiazepine use (*p* = 0.032), and mood stabilizer use (*p* = 0.031). No significant differences were found regarding age, RMT, baseline depression scores (HAMD and MDI), or stimulation protocol (all *p* > 0.050; see Table 2 for details).

**Table 2.** Baseline sociodemographic characteristics of the Regensburg patient sample.

Characteristics	Receiving augmentation		Statistics^d^
	No, *n* (%)	Yes, *n* (%)	
Total^a^	42 (34.7)	79 (65.3)	
Age^c^	45.5 (30.8–57)	48 (34–58)	*Z* = −0.743, *p* = 0.457
Sex			*p* = 0.015
Male^a^	24 (57.1)	27 (34.2)	
Female^a^	18 (42.9)	52 (65.8)	
Taking MRIs^a,e^	25 (59.5)	71 (89.9)	*p* < 0.001
Taking GABA-A PAM (Z)^a,f^	2 (4.8)	6 (7.6)	*p* = 0.712
Taking GABA-A PAM (BZD)^a,g^	2 (4.8)	15 (19.0)	*p* = 0.032
Taking GA/ICA^a,h^	3 (7.1)	18 (22.8)	*p =* 0.031
Stimulation protocol^a^			*p* = 0.056
iTBS^a^	35 (83.3)	53 (67.1)	
rTMS^a^	7 (16.7)	26 (32.9)	
Resting motor threshold^c^	41 (38–42)	43 (36–50.3)	*Z* = −1.490, *p* = 0.136
HAMD at baseline^b^	16 (13.5–19)	17 (15–21)	*Z* = −1.067, *p* = 0.286
MDI at baseline^b^	37 (31.5–41)	36.5 (31.8–42)	*Z* = −0.571, *p = 0*.568

a *n* (%).

b Median (Q1–Q3).

c Mean ± SD.

d Chi-squared test for categorical variables, Wilcoxon-test for continuous variables.

e Monoamine Reuptake Inhibitors. Includes drugs formally known as antidepressants.

f GABA-A Positive Allosteric Modulators. Includes drugs formally known as *Z*-drugs (e.g., zopiclone, zolpidem), used as hypnotics.

g GABA-A Positive Allosteric Modulators (includes drugs formally known as benzodiazepines).

h Glutamate and Ion Channel Agents. Includes drugs formally known as mood stabilizers.

### Treatment outcome and variability

#### Augsburg

**Figure 1.**
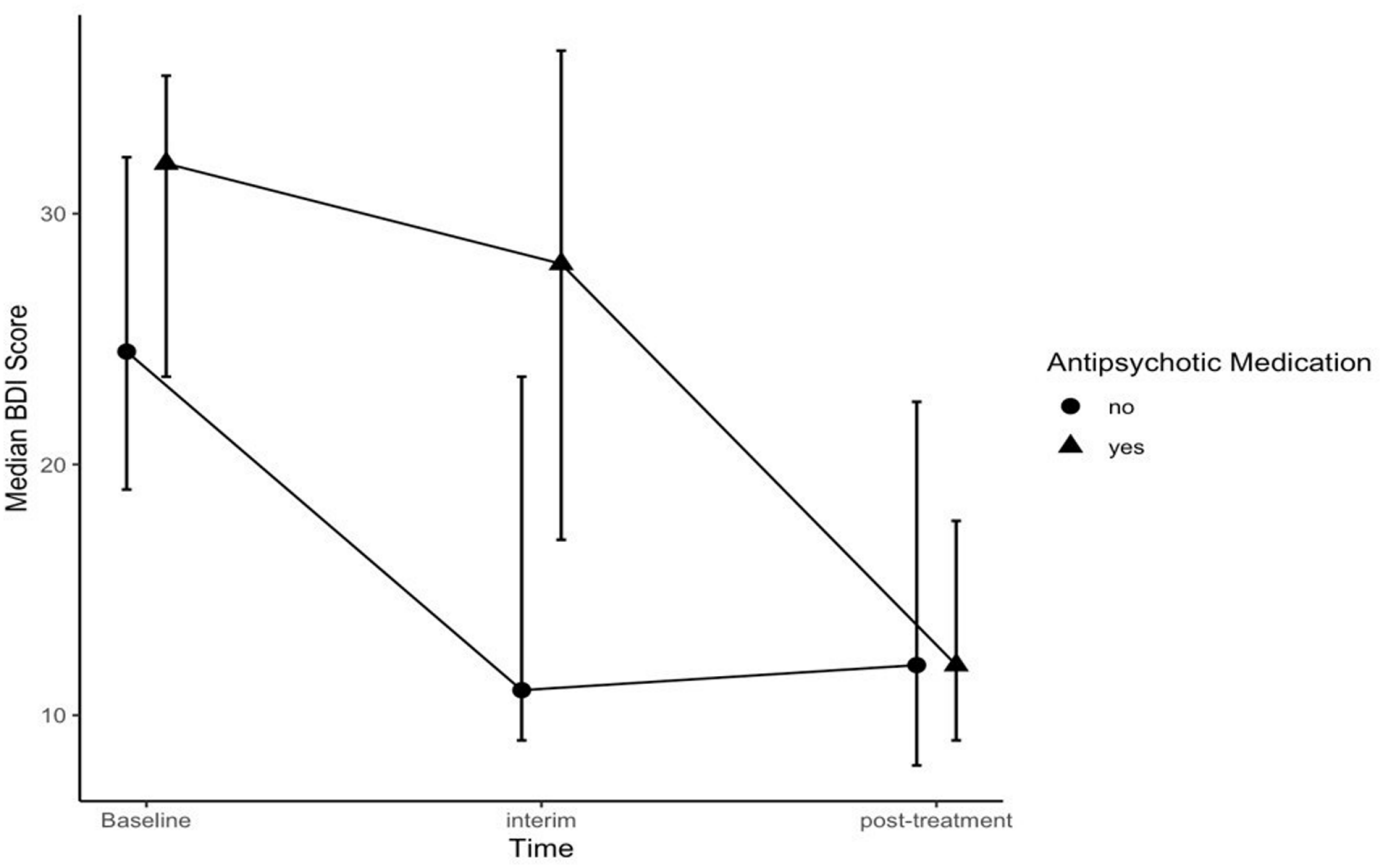
Median BDI scores over time for patients being treated with and without D2/5-HT2 receptor antagonists or D2/5-HT1A partial agonists. Error bars represent the interquartile range (IQR).

As indicated by the results of the Friedman test (χ^2^ *=* 30.18, *p* < 0.001) and the post hoc Wilcoxon signed-rank tests (T0–T1: *p*.adj < 0.001; T0–T2: *p*.adj < 0.001; T1–T2: *p*.adj < 0.001), patients’ depressive symptoms improved significantly, irrespective of their augmentation. The study was discontinued by 12 patients between T0 and T1, and by another 10 between T1 and T2. To add to the robustness of our exploratory analysis, we examined potential selection bias over time. Statistical analyses showed that between groups there was neither a statistically significant difference in the number of dropouts (T0–T1: χ^2^ = 0.50, *p* = 0.478; T1–T2: χ^2^ = 0.50, *p* = 0.478; T1–T2: χ^2^ = 2.19, *p* = 0.139) nor in improvements in BDI scores among these dropouts (*t*(11) = 1.46, *d* = 0.792, *p* = 0.173, *t*-statistic was conducted due to normal data distribution). Thus, the dropouts over time introduced no additional bias in our statistical analysis, and we decided to include the data from all patients with completed assessments at the respective time points instead of restricting the paired Mann–Whitney *U* tests to only patients with complete assessments post-treatment. These tests between groups revealed a significant difference in BDI scores between patients being treated with and without D2/5-HT2 receptor antagonists or D2/5-HT1A partial agonists 2 weeks after baseline (*r* = 0.184; *p*.adj = 0.029) but not at baseline (*r* = 0.407; *p*.adj = 0.358) or post-treatment (*r* = 0.035; *p*.adj = 0.865). To account for the between-group contrast in age, the Mann–Whitney *U* test at T1 was repeated on the residuals from a linear regression of BDI scores on age. Following this adjustment, statistical significance increased to *p*.adj = 0.015. The median scores of both groups over time are displayed in Figure 1. Due to the small sample size, no reasonable analysis could be conducted after stratification by treatment protocol. Because the groups did not differ significantly in their received protocol, we chose to report results grouped only by D2/5-HT2 receptor antagonist or D2/5-HT1A partial agonist co-medication.

**Figure 2.**
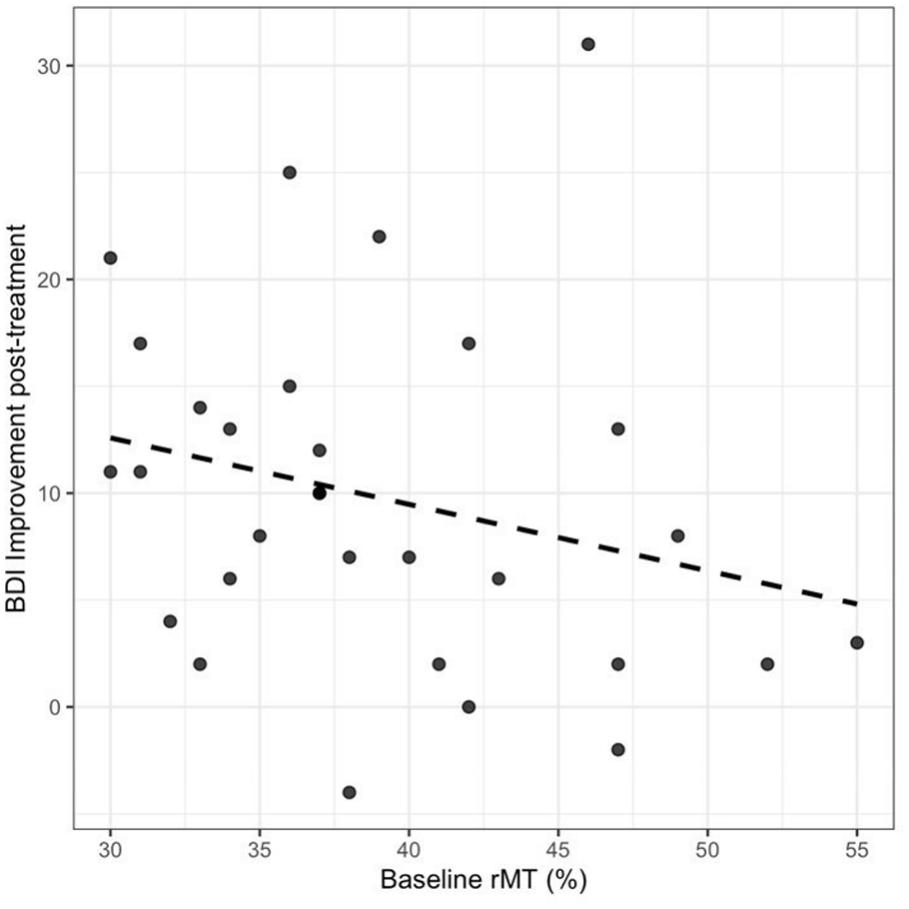
Scatterplot of baseline RMT and improvement in BDI scores.

For our secondary exploratory analysis, we investigated the correlation between the RMT and treatment outcome. For this, we chose RMT values measured at baseline as the independent variable and improvement in the BDI from baseline to post-treatment as the dependent variable. This resulted in a reduced sample size of *n* = 30, as 23 patients did not complete both 4 weeks of treatment and/or their ratings post-treatment. Our analysis showed a moderate, but nonsignificant correlation between these two variables (*r* = 0.260; *p* = 0.171). The respective scatterplot is shown in Figure 2.

#### Regensburg

Friedman tests revealed highly significant decreases in both MDI (χ²(2) = 122.40–123.39, all *p* < 0.001) and HAMD scores (χ²(2) = 113.95–115.92, all *p* < 0.001) over the course of treatment across all imputations. For the HAMD, the post hoc Wilcoxon signed-rank tests revealed significant reductions from baseline to week 3 (*Z* = −8.13 to −8.28, all *p* < 0.001), from baseline to week 6 (*Z* = −8.68 to −8.74, all *p* < 0.001), and from weeks 3 to 6 (*Z* = −4.07 to −4.17, all *p* < 0.001) across all imputed datasets. For the MDI, the post hoc Wilcoxon signed-rank tests also revealed significant reductions from baseline to week 3 (*Z* = −8.23 to −8.27, all *p* < 0.001), from baseline to week 6 (*Z* = −8.45 to −8.64, all *p* < 0.001), and from weeks 3 to 6 (*Z* = −4.74 to −4.80, all *p* < 0.001) across all imputed datasets. These results reflect a consistent and robust improvement in clinician and self-rated depressive symptoms over time, irrespective of augmentation status.

**Figure 3.**
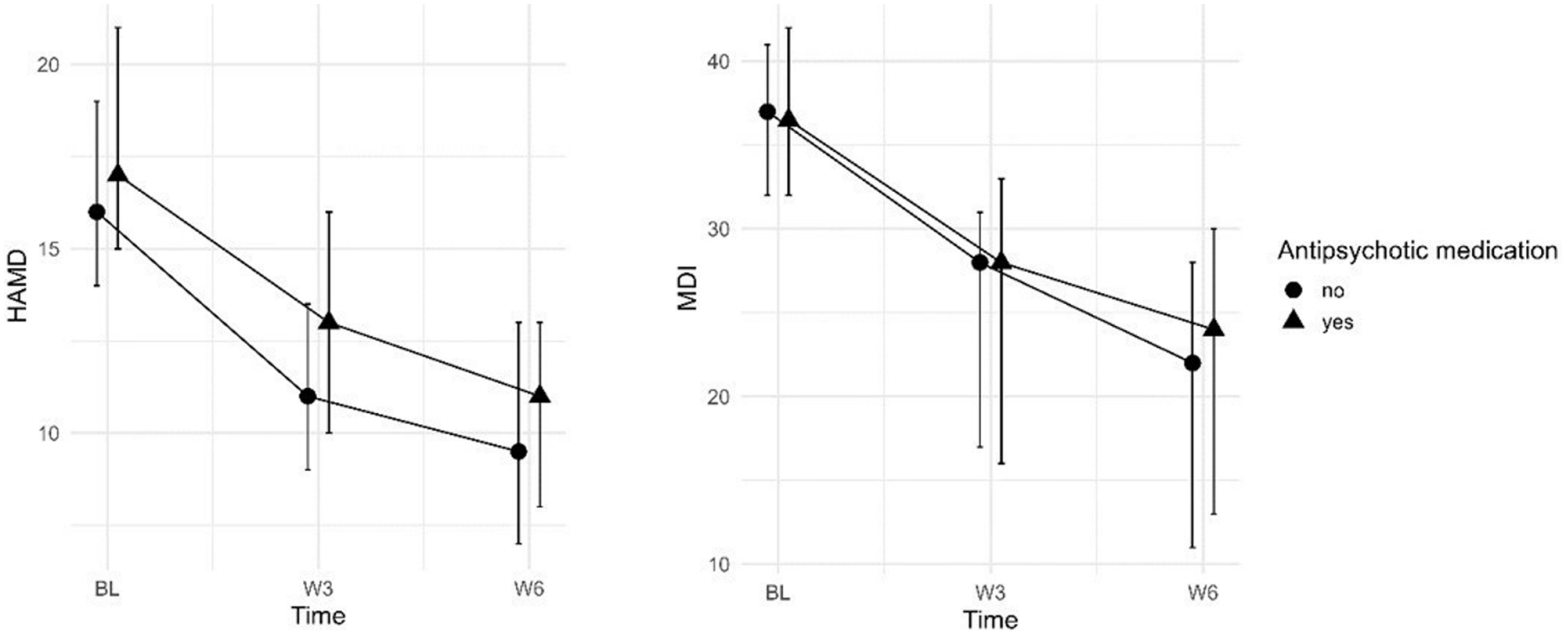
Line plots of median depressive symptom scores with IQRs for the HAMD (left panel; *n* = 106–112) and the MDI (right panel; *n* = 118–119) across the treatment period in the original (non-imputed) patient sample.

For visualization, raw score trajectories from the original data set are shown in Figure 3 for both the HAMD (left panel) and the MDI (right panel).

For both depression scales, Mann–Whitney *U* tests were conducted between patients with and without concurrent D2/5-HT2 receptor antagonists or D2/5-HT1A partial agonistsat each time point (T0*–T2*). For the MDI, no significant differences were found at baseline (*Z* = −0.56 to −0.61, *p* = 0.542–0.574), week 3 (*Z* = −0.39 to −0.62, *p* = 0.538–0.694), or week 6 (*Z* = −0.88 to −1.00, *p* = 0.319–0.379) across the imputed datasets. Likewise, for the HAMD, no significant group differences were found at baseline (*Z* = −1.07 to −1.48, *p* = 0.140–0.286), week 3 (*Z* = −1.85 to −1.91, *p* = 0.056–0.064), or week 6 (*Z* = −1.33 to −1.53, *p* = 0.127–0.182) across the imputed datasets. However, there was a descriptive trend toward higher symptom scores for patients taking D2/5-HT2 receptor antagonists or D2/5-HT1A partial agonistsirrespective of time point (see Figure 3, left panel). As only seven patients without augmentation were treated with rTMS, we chose not to further stratify results by stimulation protocol.

**Figure 4.**
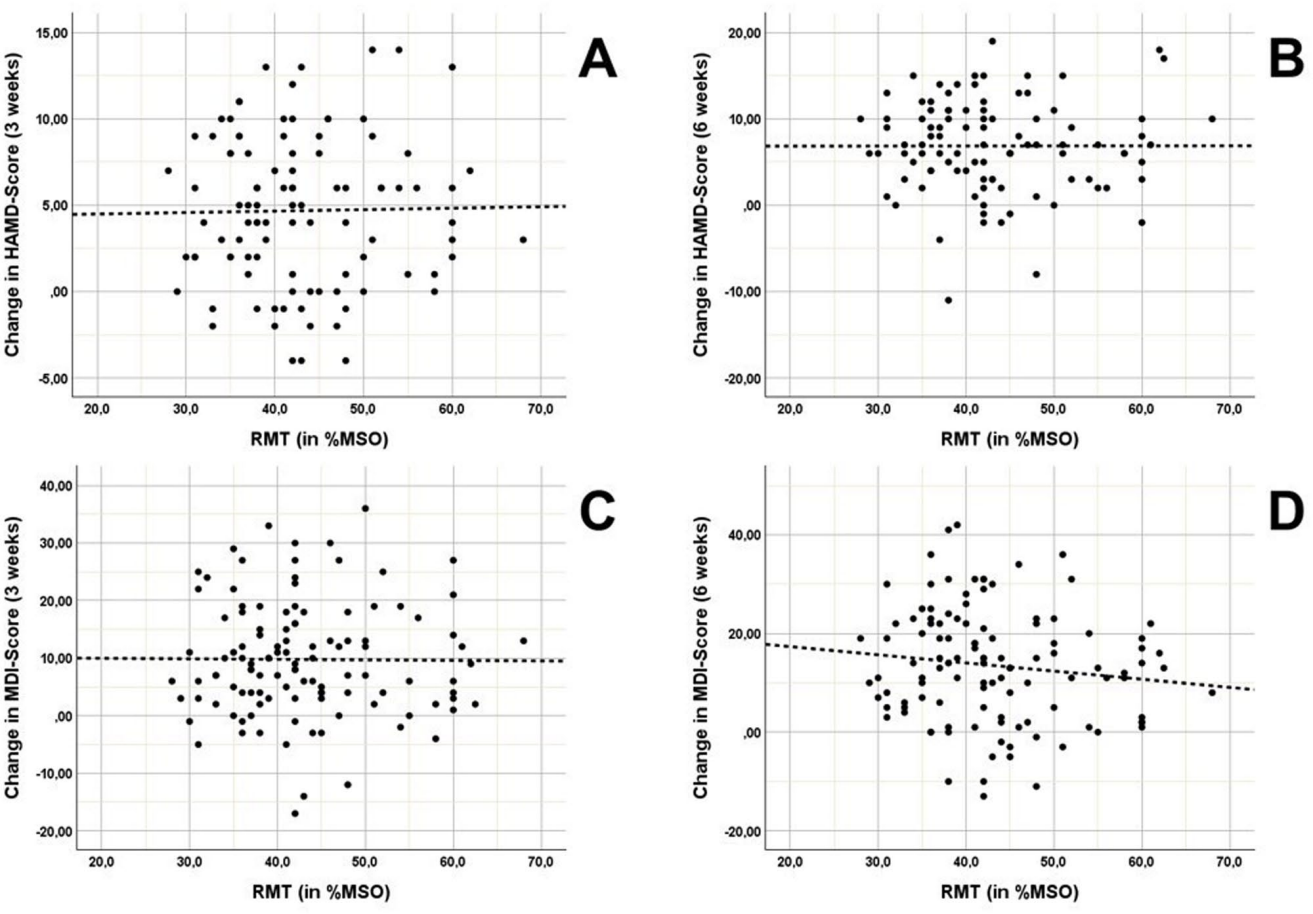
Scatterplots of baseline RMT and improvement in HAMD scores after 3 weeks (panel a) and 6 weeks (panel b) and improvement in MDI scores after 3 weeks (panel c), and 6 weeks (panel d).

For the secondary exploratory analysis, the calculated correlation coefficients analyzing the predictive association between RMT and the change in HAMD scores were negligible and statistically insignificant both for the interim rating after 3 weeks (*r =* 0.017, *p* = 0.871) and the final rating after 6 weeks of treatment (*r =* 0.001, *p* = 0.991). Likewise, the respective correlation coefficients for the MDI change scores were statistically insignificant and negligible-to-small in size both for the interim rating (*r =* −0.008, *p* = 0.931) and the final rating after 6 weeks of treatment (*r =* −0.127, *p* = 0.179). The respective scatterplots are displayed in Figure 4.

## Discussion

This study aimed to investigate the antidepressant effectiveness of TMS applications (rTMS and TBS) in patients with and without D2/5-HT2 receptor antagonist or D2/5-HT1A partial agonist co-medication and with respect to correlations to baseline RMT measures using data from two independent clinical sites. Below, we summarize the findings from each clinic separately before offering a general interpretation and highlighting the implications.

In addition to the major antidepressant effect of rTMS in the patient group from Augsburg, reflected by the significant differences in BDI values between the baseline measurement and the measurement after 4 weeks, we also observed a delayed treatment response among patients taking D2/5-HT2 receptor antagonists or D2/5-HT1A partial agonists. Patients with this co-medication still had significantly higher BDI values after 2 weeks of rTMS/iTBS treatment than patients without it, although this difference was no longer present after 4 weeks. This observation in the Augsburg sample suggests a delayed but substantial response. Although we did not formally test for a time × group interaction due to our small sample size, the descriptive pattern of delayed improvement in the augmentation group suggests a potential interaction worth further investigation in future studies using appropriate models. These results could be explained by several considerations. The Augsburg results themselves could be explained by the interaction between the medication and the rTMS treatment in the sense that D2/5-HT2 receptor antagonists or D2/5-HT1A partial agonists delayed the positive effect of rTMS. Since the antidepressant effect of rTMS is possibly due to increased dopamine release in subcortical brain regions (Cho & Strafella, 2009; Strafella et al., 2003), which in turn would be attenuated by D2/5-HT2 receptor antagonists or D2/5-HT1A partial agonists (Monte-Silva et al., 2011), this could be an approach for explaining the results. This hypothesis would be in line with both the results of Schulze et al. (2017) and those of Hebel et al. (2020). However, the differences in TMS applications (rTMS or TBS) and differences in parameters of these studies should be considered. While Hebel et al. (2020) applied standard high-frequency rTMS protocols predominantly over the left DLPFC, Schulze et al. (2016) used iTBS targeting the DMPFC, with MRI-guided coil positioning. The two protocols differ not only in stimulation site but also in pulse pattern, session duration, and likely underlying mechanisms. Additionally, the mean number of sessions and the clinical context (single-center vs multi-center, standard vs MRI-guided) varied considerably, which may further account for the divergent results regarding co-medication.

Concerning the relationship between baseline RMT and treatment response, the findings from Augsburg mirror findings in the literature. Lee et al. (2025) also reported that baseline RMT did not significantly predict rTMS/iTBS treatment outcome in a larger patient sample, which aligns with our nonsignificant correlation between RMT and BDI change.

At the Regensburg site, a parallel evaluation was conducted using both the MDI and the HAMD scores as primary outcome measures. The results of these analyses point to comparable anti-depressive effects of rTMS/iTBS irrespective of intake of D2/5-HT2 receptor antagonists or D2/5-HT1A partial agonists. In contrast to the findings from Augsburg, the Regensburg sample did not show a delayed treatment response in patients receiving D2/5-HT2 receptor antagonists or D2/5-HT1A partial agonists. Several sample characteristics may account for this discrepancy. First, the overall older age and more widespread use of psychopharmacological co-medication—particularly among patients receiving D2/5-HT2 receptor antagonists or D2/5-HT1A partial agonists—suggest a higher degree of polypharmacy. This includes significantly more frequent intake of antidepressants, benzodiazepines, and mood stabilizers. The antidepressant effects of rTMS/iTBS in these patients might have been supported or amplified by additive or synergistic drug effects, potentially masking or offsetting any negative interaction between D2/5-HT2 receptor antagonists or D2/5-HT1A partial agonists and rTMS mechanisms. Additionally, the heterogeneity of agents (and a missing stratification by receptor antagonism and partial agonism) used in the sample may also explain the lack of delayed treatment response. Unlike previous studies (e.g., Hebel et al., 2020), patients received 6-weeks rTMS treatment with measurements at weeks 3 and 6, which may have further contributed to the normalization of response trajectories across medication groups. Moreover, a potential selection bias has to be considered, as data come from a retrospective analysis of a naturalistic sample and patients, who experienced an early response and stopped treatment after 3 weeks were not included in the analysis. Finally, self-assessments such as BDI and MDI versus interview-based depression assessments such as HAMD may vary considerably in terms of the extent to which changes in depression are perceived subjectively or externally. All these methodological differences may account for the differences in the observed results.

Finally, and similarly to the Augsburg sample, we did not find a statistically significant relationship between RMT at baseline and treatment response in the Regensburg sample.

Taken together, our findings from both sites at Augsburg and Regensburg add robustness to the results reported by Lee et al. (2025) regarding the relationship between RMT and treatment response, as this observation has now been replicated in populations differing in sample size and several characteristics such as location, co-medication, age and sex. Despite measuring treatment response with three different rating scales, none of our findings significantly deviated from each other. Given the variability in reported response rates to rTMS/iTBS due to the use of different rating scales (Leuchter et al., 2023), this further reinforces the hypothesis that RMT has no substantial effect on treatment response.

Taken together, both datasets support the general antidepressant efficacy of rTMS and iTBS in patients with moderate to severe depressive symptoms, including those receiving D2/5-HT2 receptor antagonists or D2/5-HT1A partial agonists. While the Augsburg data suggest a delayed response in the augmentation group, Regensburg did not find indications for a delayed response in this subgroup of patients. These discrepancies may be attributable to differences in outcome measures (BDI vs MDI/HAMD), statistical power, sample composition, sample selection, treatment duration, data sampling timepoints, or clinical setting. The results extend the findings from previous studies. Hebel et al. (2020) reported a lower efficacy of rTMS in patients treated with D2/5-HT2 receptor antagonists or D2/5-HT1A partial agonists, while Schulze et al. (2017) did not find this. Our data suggest that the timing of symptom improvement may be a critical factor. A delayed antidepressant effect could explain the reduced efficacy observed in shorter or less intensive treatment protocols (Hebel et al., 2020), while longer treatment durations (Schulze et al., 2017) may capture the full therapeutic benefit even in this subgroup. Regardless of the explanatory approach, the present results highlight the importance of a treatment duration of at least 4 weeks, especially for people taking D2/5-HT2 receptor antagonists or D2/5-HT1A partial agonists, and emphasize the importance of patience in treatment with rTMS for moderate to severe depressive symptoms. A premature discontinuation of treatment due to an avoidable lack of effects could lead to a lack of significant antidepressant effects, which would have been potentially possible with rTMS as a treatment method.

Both datasets share important limitations. As this is a retrospective registry evaluation, there were no sham treatment groups with which our results could have been compared. Due to parallel (complementary) therapies, different medication and the inclusion of both out-patients and inpatients, the effects cannot necessarily be attributed solely to D2/5-HT2 receptor antagonists, D2/5-HT1A partial agonists or rTMS treatment. Potential confounding variables cannot be ruled out due to the lack of randomization and a comparison group without rTMS treatment. Such accompanying research in a naturalistic setting increases external validity, but at the same time comes with limitations in internal validity. Nevertheless, replications with larger samples and prospective research with controlled conditions and control groups are of great importance with regard to the transferability of our results. As highlighted by Schulze et al. (2017), conducting prospective randomized studies on the former would be difficult to justify, given preclinical evidence suggesting harmful effects of psychotropic medications, which in turn may underscore the value of retrospective naturalistic analyses despite their inherent limitations. Retrospective evaluations at different study sites also result in differences in important parameters (measurements at 2 and 4 vs at 3 and 6 weeks, different survey instruments).

Another limitation concerns the pharmacological heterogeneity. Differences in receptor profiles, especially between full dopaminergic antagonists and partial agonists, may have influenced treatment outcomes in complex and opposing ways. Partial agonists such as aripiprazole may have attenuated potential dampening effects observed with full antagonists such as risperidone. Our analysis could—due to the lack of respective data entries—not stratify results by these mechanisms of action, dosage equivalence, or what exactly indicated the prescription in each patient’s specific case. Further, confounding effects related to concomitant medication (see Table 1) could not be statistically controlled, which warrants careful consideration in future research employing a larger and more homogeneous sample. Hence, future studies could usefully implement respective data collection to answer these research issues in real-world datasets and might consider analyzing D2/5-HT2 receptor antagonists and D2/5-HT1A partial agonists separately in the context of outstanding questions with respect to the impact of dopaminergic neuromodulation on TMS response. Despite these limitations, the study has notable strengths. It provides rare comparative data across two independent clinical sites using real-world patient populations, and it highlights the potential temporal dynamics of treatment response in patients with complex medication profiles. These findings may help clinicians better anticipate treatment trajectories and set more realistic expectations when using rTMS in combination with D2/5-HT2 receptor antagonists or D2/5-HT1A partial agonists.

## Conclusions

Our results support the notion that both concurrent treatment with D2/5-HT2 receptor antagonists or D2/5-HT1A partial agonists and RMT do not substantially impact the effectiveness of TMS treatment in clinical settings. Further, we observed a delayed response to TMS treatments at one site, thereby posing the question whether TMS paradigms might need more than 4 weeks of treatment in some cases of major depressive disorder. Further research is needed to investigate potential underpinnings at the neurobiological level. The combination of medication or other forms of therapy with TMS and their interactions requires further attention in research, both in real-world data analyses as well as controlled studies.

## Supplemental Material

sj-docx-1-jop-10.1177_02698811251413510 – Supplemental material for Antipsychotic co-medication and treatment response to rTMS and iTBS in depression: Data from clinical records from two independent clinical sites Supplemental material, sj-docx-1-jop-10.1177_02698811251413510 for Antipsychotic co-medication and treatment response to rTMS and iTBS in depression: Data from clinical records from two independent clinical sites by Amelie Völkel, Joshua Tritsch, Marco Poli, Katharina Kerkel, Alkomiet Hasan, Berthold Langguth, Andreas Reissmann and Wolfgang Strube in Journal of Psychopharmacology

## Notes

1. One patient received only 1000 pulses per session, with otherwise identical rTMS protocol parameters.
