# Supplementary material for: Antipsychotic co-medication and treatment response to rTMS and iTBS in depression: Data from clinical records from two independent clinical sites
Source: J Psychopharmacol. 2026 Feb 23;40(4):595–605. doi: 10.1177/02698811251413510 (PMC13283503; doi:10.1177/02698811251413510)
Supplement: sj-docx-1-jop-10.1177_02698811251413510 – Supplemental material for Antipsychotic co-medication and treatment response to rTMS and iTBS in depression: Data from clinical records from two independent clinical sites [file sj-docx-1-jop-10.1177_02698811251413510.docx]

## **Supplementary Note:**

As described, the data set was based on existing register data at the time of evaluation. Some of the BDI questionnaires, which were completed by hand, were not complete. If a questionnaire was completed >80%, the mean values were used for interpolation.

Different versions of the BDI were used in some cases in the evaluated data set. 5 people out of 65 patients therefore completed a different version than the remaining 60. 3 of the 5 people had data for all 3 measurement times. As a result of the lack of comparability of the two versions due to different cut-off values:

BDI-I: 0-10 no to minimal depression

10-18 mild to moderate depression

19-29 moderate depression

30-63 severe depression

BDI-II: 0-13 no to minimal depression

14-19 mild to moderate depression

20-28 moderate depression

29-63 severe depression

the calculation of delta values was not an option and the data analysis are therefore based on the 60 people who all completed the same version of the BDI.
